# Spatio-temporal prediction and reconstruction network for video anomaly detection

**DOI:** 10.1371/journal.pone.0265564

**Published:** 2022-05-26

**Authors:** Ting Liu, Chengqing Zhang, Xiaodong Niu, Liming Wang

**Affiliations:** 1 State Key Lab for Electronic Testing Technology, North University of China, Taiyuan, 030051, China; 2 College of Mechatronics Engineering, North University of China, Tai Yuan, 030051, China; Institut de Robotica i Informatica Industrial, SPAIN

## Abstract

The existing anomaly detection methods can be divided into two popular models based on reconstruction or future frame prediction. Due to the strong learning capacity, reconstruction approach can hardly generate significant reconstruction errors for anomalies, whereas future frame prediction approach is sensitive to noise in complicated scenarios. Therefore, a solution has been proposed by balancing the merits and demerits of the two models. However, most methods relied on single-scale information to capture spatial features and lacked temporal continuity between the video frames, affecting anomaly detection accuracy. Thus, we propose a novel method to improve anomaly detection performance. Because of the objects of various scales in each video, we select different receptive fields to extract comprehensive spatial features by the hybrid dilated convolution (HDC) module. Meanwhile, the deeper bidirectional convolutional long short-term memory (DB-ConvLSTM) module can remember the temporal information between the consecutive frames. Experiments prove that our method can detect abnormalities in various video scenes more accurately than the state-of-the-art methods in the anomaly-detection task.

## Introduction

In recent years, anomaly detection in surveillance videos has become a crucial research task due to its potential application value for smart cities and public security [1]. Traditional surveillance systems depend on artificial means to recognize abnormalities in the massive amount of real-time video data. This way increases working hours, labor requirements, and error rate. Hence, automatic detection of abnormal events [2] has drawn more and more attention from researchers. The intelligent surveillance system is a video supervising technology that uses an automatic video analysis algorithm to find abnormal behaviors as soon as possible. However, anomaly detection is subject to certain limitations so far. First, the abnormal events are much fewer than normal samples in complex video surveillance data. Second, there is no standard definition of “abnormality” because of context-dependent and human-defined semantics anomalous samples. Therefore, popular supervised methods are not suitable for our anomaly detection task.

Most state-of-the-art methods [3,4] usually employ unsupervised technologies that use normal events to train the network model. The abnormal events are detected as significant deviations from the learned model. In particular, many approaches use reconstruction error-based methods [5,6], which train the normal samples and generate frames as consistently as possible with the normal samples. Regular activities produce a small reconstruction error when testing the learned model, whereas abnormal movements cause a relatively large error. Nevertheless, obtaining a significant reconstruction error for anomalies is challenging due to a deep neural network’s high learning capacity and generalization ability. Furthermore, the methods recognize abnormalities regardless of context information and lack temporal continuity owing to the self-reconstructed generated frames. Therefore, it is accessible to the missed and false detection phenomena while running these methods.

Considering the disadvantages of reconstruction methods, the video-prediction algorithms [7,8] have been verified more efficient for anomaly detection. By only training regular events to obtain a prediction model, the prediction methods follow the rule that normal events are predictable, whereas abnormal events are unpredictable. It can make up for the shortcomings of reconstruction methods, making normal and abnormal behaviors more distinguishable. However, the traditional future-frame prediction model heavily depends on the information of former frames, which is quite sensitive to any changes of these frames.

To solve the problems mentioned above, a new idea is proposed by considering the advantages and disadvantages of prediction and reconstruction methods [9,10]. The future frame prediction model expands the reconstruction error of abnormalities, making it easier to distinguish abnormal events. At the same time, the reconstruction model enhances the ability to predict future frames from regular events, which ensures robustness to noise. Nevertheless, the literature [9] acquired only single-scale information from the previous layer based on a spatio-temporal AutoEncoder (STAE), leading to the loss of detailed information for objects of different sizes. The literature [10] used double conventional U-Net to integrate prediction and reconstruction network (IPR) for anomaly detection. Still, this method cannot fully consider the motion continuity between the video frames.

Motivated by the aforementioned anomaly detection task, it is necessary to sufficiently consider multi-scale features and spatiotemporal continuity, which are essential for recognising abnormal behaviours. Recently, lots of works have achieved great detection performance by using multi-scale features of images [11,12]. Owing to the camera position and angle, objects multi-scale features extraction can effectively improve the performance of target detection. This paper proposes a novel spatio-temporal prediction and reconstruction network, i.e., STPR-net, which integrates the multi-scale spatial features and temporal information. In the prediction part, starting from the second downsampling of U-Net, we use the HDC module [13] to extract multi-scale spatial features and learn the object’s scale variations. Then, at the end of the encoding path of U-Net, we adopt the DB-ConvLSTM [14] to handle the temporal information and obtain the complex motions between the continuous video frames. In the reconstruction part, we use newly designed AutoEncoder (AE) structure to reconstruct the future frame through the space and time dimension, which effectively improves the accuracy of the prediction results.

The rest of this article is organized as follows. Section 2 reviews the related works of anomaly detection. Section 3 presents the entire model framework of our method. Section 4 illustrates and discusses the experimental evaluation through a series of public datasets. Finally, Section 5 summarises the paper and points out the future study directions.

## Related work

With the rapid development of deep-learning technology, it has apparent advantages in anomaly detection tasks. Among all existing methods, the idea of reconstruction or future frame prediction plays a vital role in detecting anomalies.

### Reconstruction methods

Recently, due to the strong capability of deep-learning networks in reconstruction, it has undoubtedly made progress in anomaly detection task. Specifically, Zhai et al. [5] created a deep-structured energy-based model to detect anomalous events. Hasan et al. [6] obtained a regular model with the normal video sequences based on the AE structure and then applied it to identify the irregularities. These researches indicated that convolution is mainly used to extract features, so this structure hardly encodes temporal dependencies in a long video sequence. Consequently, Chong et al. [15] and Luo et al. [16] presented convolutional long short-term memory (ConvLSTM) layers to model temporal information. Li et al. [17] proposed the multivariate Gaussian fully convolution adversarial autoencoder (MGFC-AAE) to detect anomalies by considering gradient and optical flow patches. George et al. [18] used a non-uniform spatio-temporal region resembling parallelepipeds to extract the related histogram features. These methods simultaneously consider normal appearance and motion features from the input data, further boosting the performance for video analysis.

### Prediction methods

Inspired by the fact that future frame prediction has achieved outstanding results in the field of computer vision, the prediction model aims to use the difference between the predicted frame and its ground truth to detect abnormal events. For example, Munawar et al. [7] built a deep prediction model to see the abnormal operation of industrial robots. Villegas et al. [8] combinedthe LSTM network with analogy-based AE to settle long-term video-prediction matters. Additionally, Liu et al. [19] proposed an approach to predict future frames based on U-Net, which relies on the skip connection to obtain the essential structural characteristics between high-level and low-level layers. However, these prediction methods have a typical problem of poor anti-noise capability. Based on the previous works [9,10], we connect the prediction and reconstruction module in series to improve the anomaly detection performance.

## Proposed method

### The framework of our method

**Fig 1:**
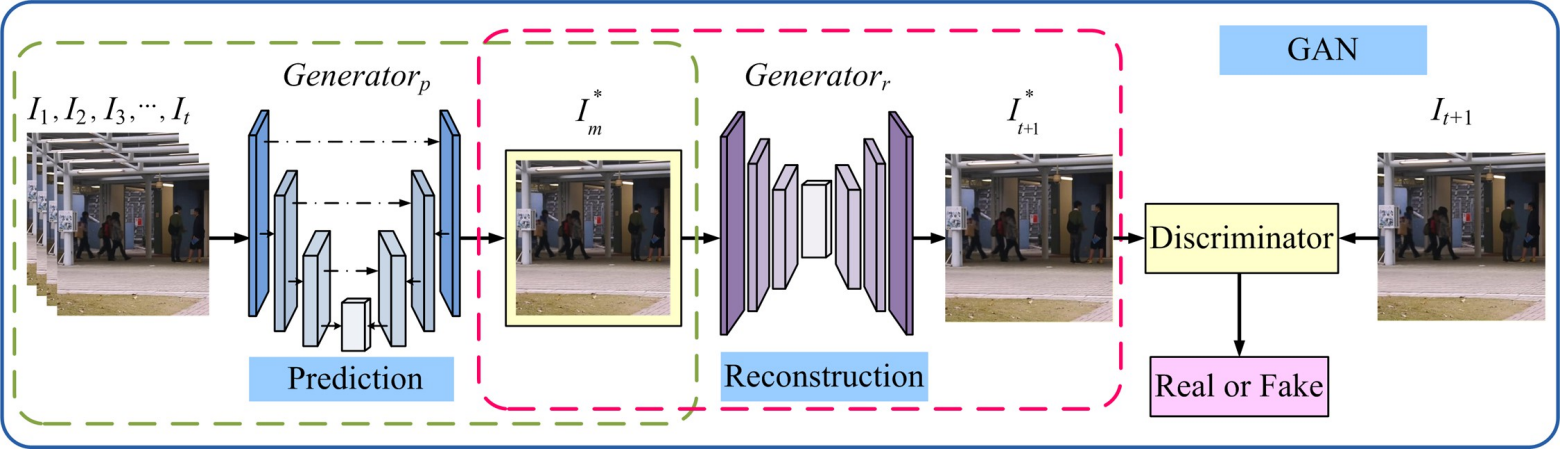
Overall framework of our method. The framework is mainly composed of prediction module, reconstruction module, and GAN module.

The overall framework of our method is displayed in Fig 1. The architecture comprises three parts: the prediction module, the reconstruction module, and the generative adversarial network (GAN) module. Unlike the study of Tang et al. [10], our network inputs T continuous frames into the predictive module one by one, achieving effective fusion of multi-scale spatial features and temporal information. To enhance the robustness to noise of the predicted frames, we add a reconstruction module into our network after the prediction module. The reconstruction module uses an AE structure to retain the multi-scale spatiotemporal distribution information of the predicted frames, improving the prediction ability from normal events. Meanwhile, we also adopt the GAN module consisting of a generator network (G) and a discriminative network (D) to optimise our network through various loss functions. The different parts of the proposed framework are illustrated in the next.

#### Prediction module

**Fig 2:**
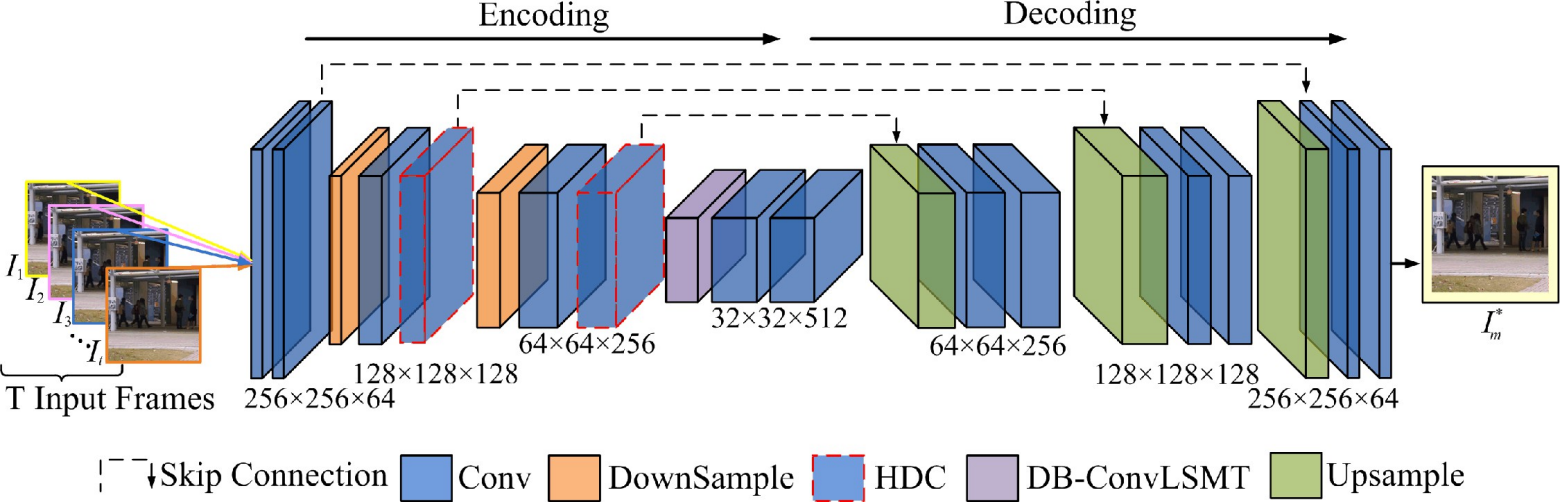
Structure of prediction module. The input and output size of the module are both 256 × 256 × 3. The kernel sizes of all convolution and deconvolution are set to 3 × 3 and the downsample layers are set to 2 × 2.The resolutions of feature maps are equal inthe same convolution layer.

Fig 2 presents the details of the prediction module. The module comprises an encoding path and a decoding path. We insert an HDC network to capture multi-scale spatial features of the training data and then adopt DB-ConvLSTM to model temporal information between the consecutive T frames in a nonlinear way.

Due to the different positions and angles of the camera, the forms and sizes of objects are significantly different. Recently, the HDC network can successfully tackle the multi-scale feature extraction task to benefit from the spatial feature information of things. At the same time, the detailed spatial information tends to lose partly due to the downsampling of the U-Net structure. To improve the representative capacity of the whole model, first, the proposed network can extract multi-scale spatial information; second, it can make up for the detailed information loss because of the downsampling operation. Therefore, we add the HDC network starting from the second downsampling layer of the U-Net to capture the features as detailed as possible. The previous study shows that the convolution before first downsampling will not cause loss to feature data.

**Fig 3:**
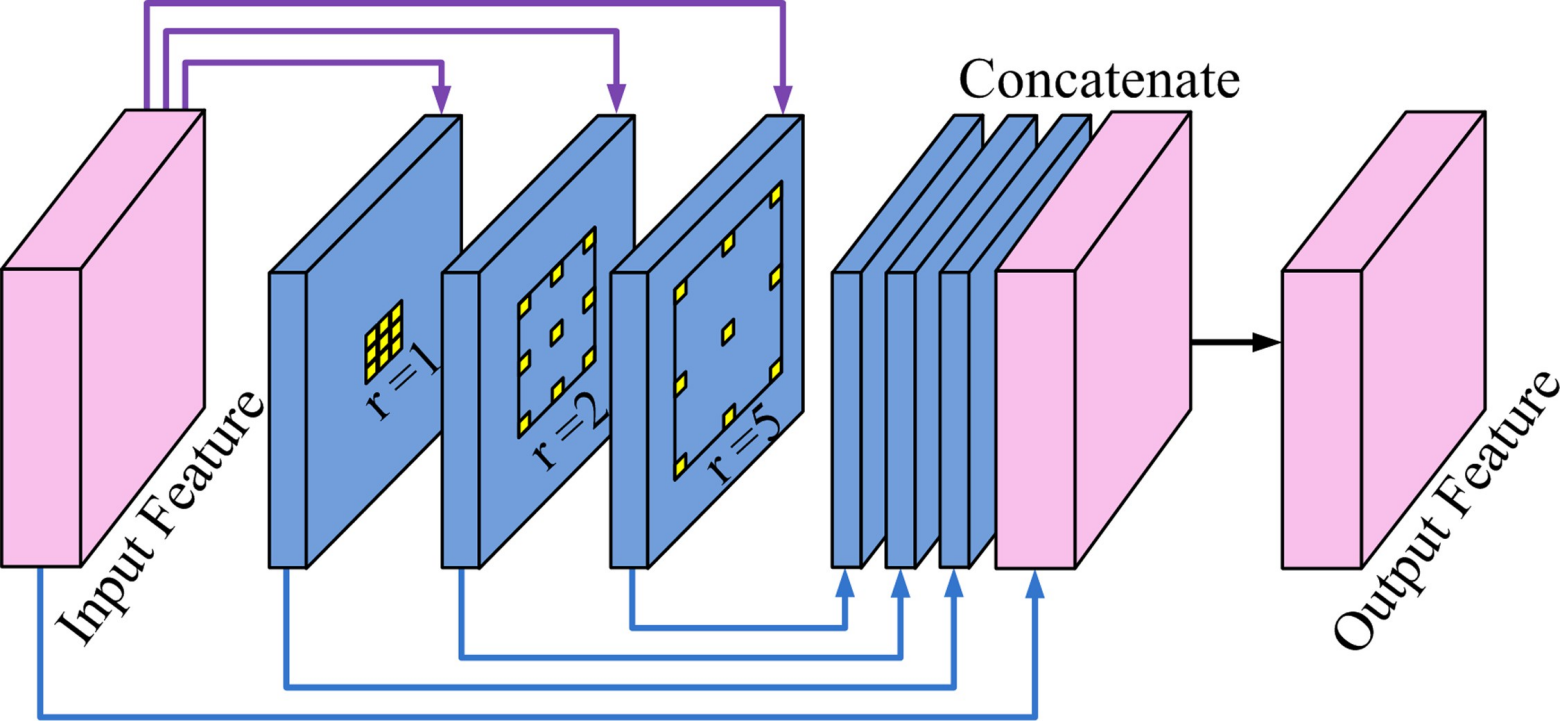
Structure of HDC module. The sizes of input feature maps are 128 × 128 × 128 and 64 × 64 × 256 successively. The dilation rates set to 1, 2, and 5, respectively.

The structure of HDC is presented in Fig 3. The input feature data are sent into three different model streams. These streams can obtain different receptive field sizes and extract multi-scale features using a set of dilated convolution with varying dilation rates. To the best of our knowledge, a low dilation rate seems suitable for capturing features of small objects, while a high dilation rate is fit for big things. Finally, the feature maps from each stream are concatenated with the input feature data for comprehensively considering multi-scale spatial features information.

The anomaly detection methods commonly use three-dimensional (3D) convolution or ConvLSTM [20] for time modeling of the input data. The 3D convolution needs more time to calculate the model parameters. Therefore, some methods adopt ConvLSTM to extract the temporal features. However, the ConvLSTM can only process the sequence data forward. According to the researches [14,21], it is also worth mentioning that considering both forward and backward information is complementary to capture temporal correlation features for predicting future frames. Thus, the proposed model leverages the DB-ConvLSTM module to obtain the related temporal information between the video frames.

**Fig 4:**
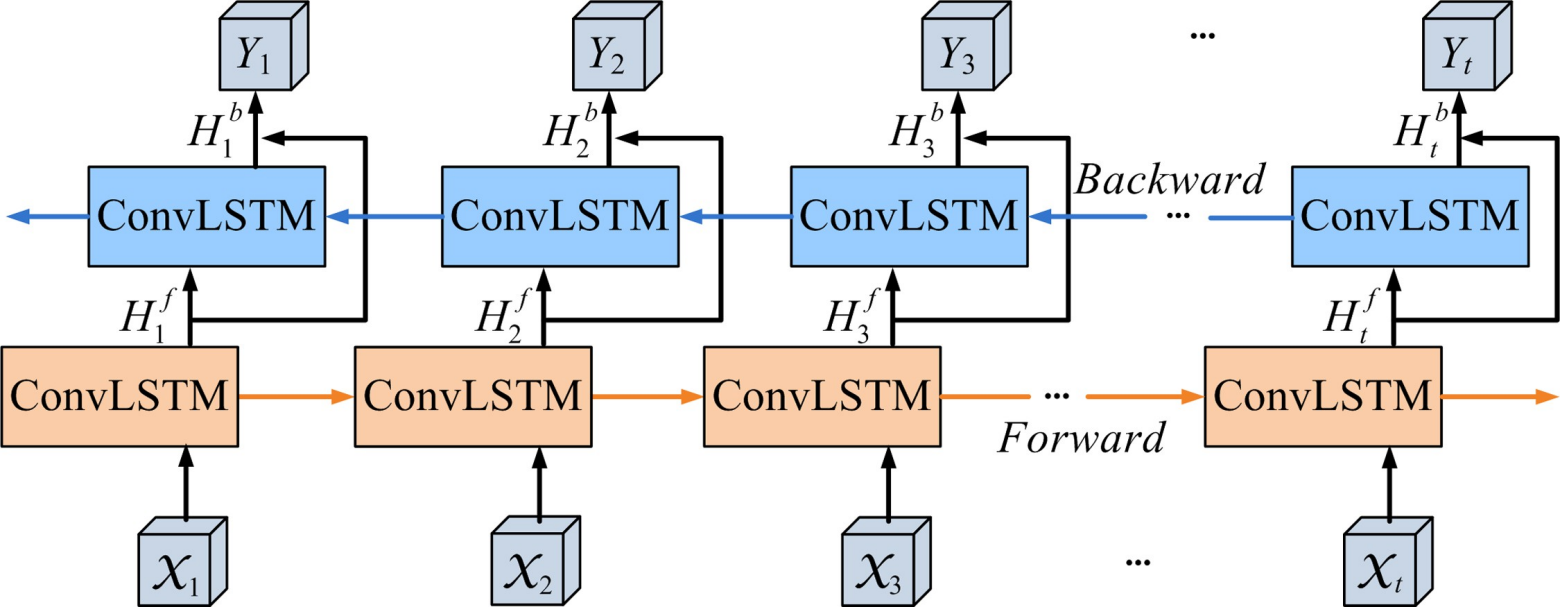
Structure of DB-ConvLSTM module.

The input pattern of our model is different from the current methods that stack T sequential frames together into the model. Among these methods, the T frames are linked to each corresponding channel in the first output feature data, resulting in the collapse of temporal information [22]. Thus, we feed T frames into the encoder orderly to generate corresponding feature maps. The DB-ConvLSTM comprises a shallow forward and a deeper backward layer (see Fig 4). More specifically, {$H_{t}^{f}$} represents the related outputs of forwarding sequential feature maps from the ConvLSTM units in the forward layer. The deeper backward layer takes over the forward sequential results {$H_{t}^{f}$} to generate {$H_{t}^{b}$}corresponding outputs of back sequential feature maps. And then, we use Eq (1) to simultaneously handle the forward and the backward features data to get the final output sequence {*Y*_*t*_}. Finally, the feature information can exchange between the forward and backward layer to extract more detailed and complementary spatiotemporal features. As shown in Fig 4, we send the final output *Y*_*t*_ into the decoding process.


$$Y_{t}=\text{tanh}(W_{y}^{H^{f}}*H_{t}^{f}+W_{y}^{H^{b}}*H_{y}^{b}+b)$$ (1)

#### Reconstruction module

**Fig 5:**
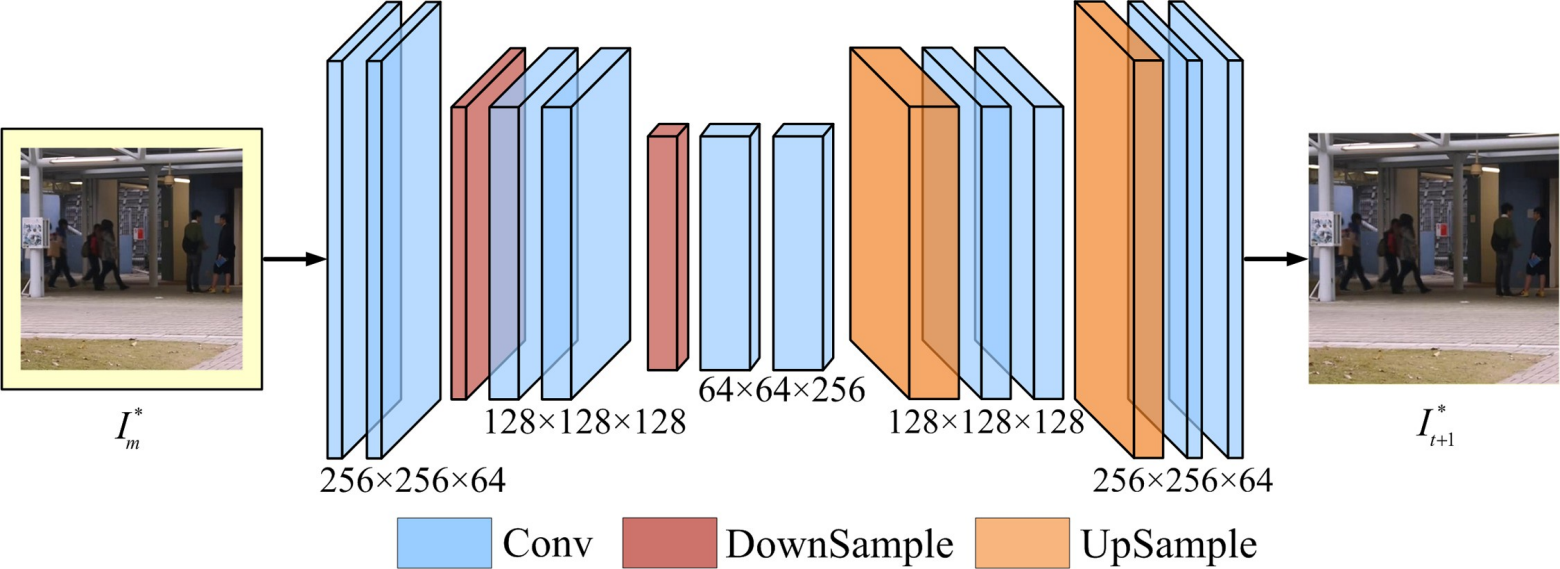
Structure of reconstruction module. The input and output size of the module are both 256 × 256 × 3. The kernel sizes of all convolution and deconvolution are set to 3 × 3 and the downsample layers are set to 2 × 2. The resolutions of feature maps are equal in the same convolution layer.

As shown in Fig 5, we use the newly designed AE structure to reconstruct the predicted frame $I_{t+1}^{*}$ from the intermediate frame $I_{m}^{*}$. Subsequently, we adopt a series of objective constraints function to optimise the proposed network, making $I_{t+1}^{*}$ closer to *I*_*t*+1_.

#### GAN module

The GAN module leverages the G and D to optimize alternately during the training phase, fully capturing the data distribution. The G aims to generate future frames as realistic as possible, whereas D attempts to identify the frames generated by G. We use the STPR-net as G, then order(*I*_1_, *I*_2_, *I*_3_, …, *I*_*t*_) frames before the current frame *I*_*t*+1_ as the input tensor, and the generated frame $I_{t+1}^{*}$ as the output tensor. For D, we choose PatchGAN [23] to strengthen the ability to distinguish the difference between the genuine frame and generated frame, guiding our model to focus attention on local image patches features.

### Loss function

We employ spatial and temporal loss functions to optimize the proposed method and minimize the gap between the generated frame and its ground truth. More specifically, the intensity loss can ensure the similarity of all pixels in the whole RGB space, and the gradient loss can retain the sharpness of the generated images. Therefore, we use intensity and gradient loss as the spatial constraint to make the generated frame I* identify with the corresponding ground truth *I*. The intensity loss and gradient loss are calculated as

$$L_{int}(I^{*},I)={\left\|I^{*}-I\right\|}_{2}^{2}$$ (2)

$$L_{gd}(I^{*},I)=\sum_{i,j}{\left\|\left|I_{i,j}^{*}-I_{i-1,j}^{*}|-|I_{i,j}-I_{i-1,\;j}\right|\right\|}_{1}+{\left\|\left|I_{i,j}^{*}-I_{i,j-1}^{*}|-|I_{i,j}-I_{i,j-1}\right|\right\|}_{1}$$ (3)

Moreover, the previous researches [24,25] indicated that the RGB difference could take the place of the optical flow [26] as an effective temporal constrain. This constrain can reach a similar effect but significantly reduce the running time. The temporal loss is defined as follows:

$$L_{rgb}(I^{*},I)={\left\|\left|I_{t+1}^{*}-I_{t}|-|I_{t+1}-I_{t}\right|\right\|}_{1}$$ (4)

We also leveraged GAN to constrain the training process owing to its excellent image generation [27] and video-prediction [10] performance in recent years. Specifically, G attempts to generate future frames that are as realistic as possible, whereas D aims to distinguish the frames generated by G. Ideally, the goal of the GAN is to reach the Nash equilibrium. When constraining the D, the network aims to classify I* into class 0 and *I* into class 1, where 0 indicates the generated frame, and 1 represents the genuine frame. When optimizing the G, the process is to make the generated frames I* classified into class 1 by D. The adversarial loss functions for D and G are defined as

$$L_{adv}^{D}(I^{*},I)=\frac{1}{2}{\left(D(I^{*})-0\right)}^{2}+\frac{1}{2}{\left(D(I)-1\right)}^{2}$$ (5)

$$L_{adv}^{G}(I^{*})=\frac{1}{2}{\left(D(I^{*})-1\right)}^{2}$$ (6)

To acquire a well-trained model with a better ability to detect abnormalities, we collect all the constraints above, i.e., spatial loss, temporal loss, and adversarial loss, for the final objective optimization functions as follows:

$$L_{G}=\alpha_{int}L_{int}+\alpha_{gd}L_{gd}+\alpha_{rgb}L_{rgb}+\alpha_{adv}+L_{adv}^{G},$$ (7)

$$L_{D}=L_{adv}^{D}$$ (8)

where *α*_*int*_, *α*_*gd*_, *α*_*rgb*_, and *α*_*adv*_ are coefficients for the corresponding loss functions, respectively.

### Anomaly detection

As far as we know, Peak Signal to Noise Ratio (PSNR) [28] is often picked to evaluate the image quality. After obtaining the well-trained model, we calculate the difference between the generated frame I* and corresponding ground truth *I* for anomaly detection.

$$PSNR(I^{*},I)=10log_{10}\frac{{\left[\max_{I^{*}}\right]}^{2}}{\frac{1}{N}\sum_{i=0}^{N}{\left(I_{i}^{*}-I_{i}\right)}^{2}}$$ (9)

where max_I*_ denotes the maximum value of the image pixels, *N* represents the total number of pixels, and *i* is the pixel index.

We use the PSNR values to assess the generated frames in the test process. A higher PSNR indicates that the generated frame resembles its ground truth. It will be detected as a regular event and vice versa. For comparison, the PSNR values of all frames are normalized to the range of [0, 1] in each test video. The regular score is expressed as

$$S(t)=\frac{PSNR(I_{t}^{*},I_{t})-\min_{t}PSNR(I_{t}^{*},I_{t})}{\max_{t}PSNR(I_{t}^{*},I_{t})-\min_{t}PSNR(I_{t}^{*},I_{t})}$$ (10)

where the min_*t*_*PSNR* is the minimum value of the PSNR in every test video frame and the max_*t*_*PSNR* is corresponding maximum value.

## Experimental results and discussion

This section has analyzed the proposed method performance on the Chinese University of Hong Kong (CUHK) Avenue dataset [29] and the University of California San Diego (UCSD) Pedestrian dataset [30]. The entire model was trained using TensorFlow with an NVIDIA Tesla V100.

### Evaluation metric

**Fig 6:**
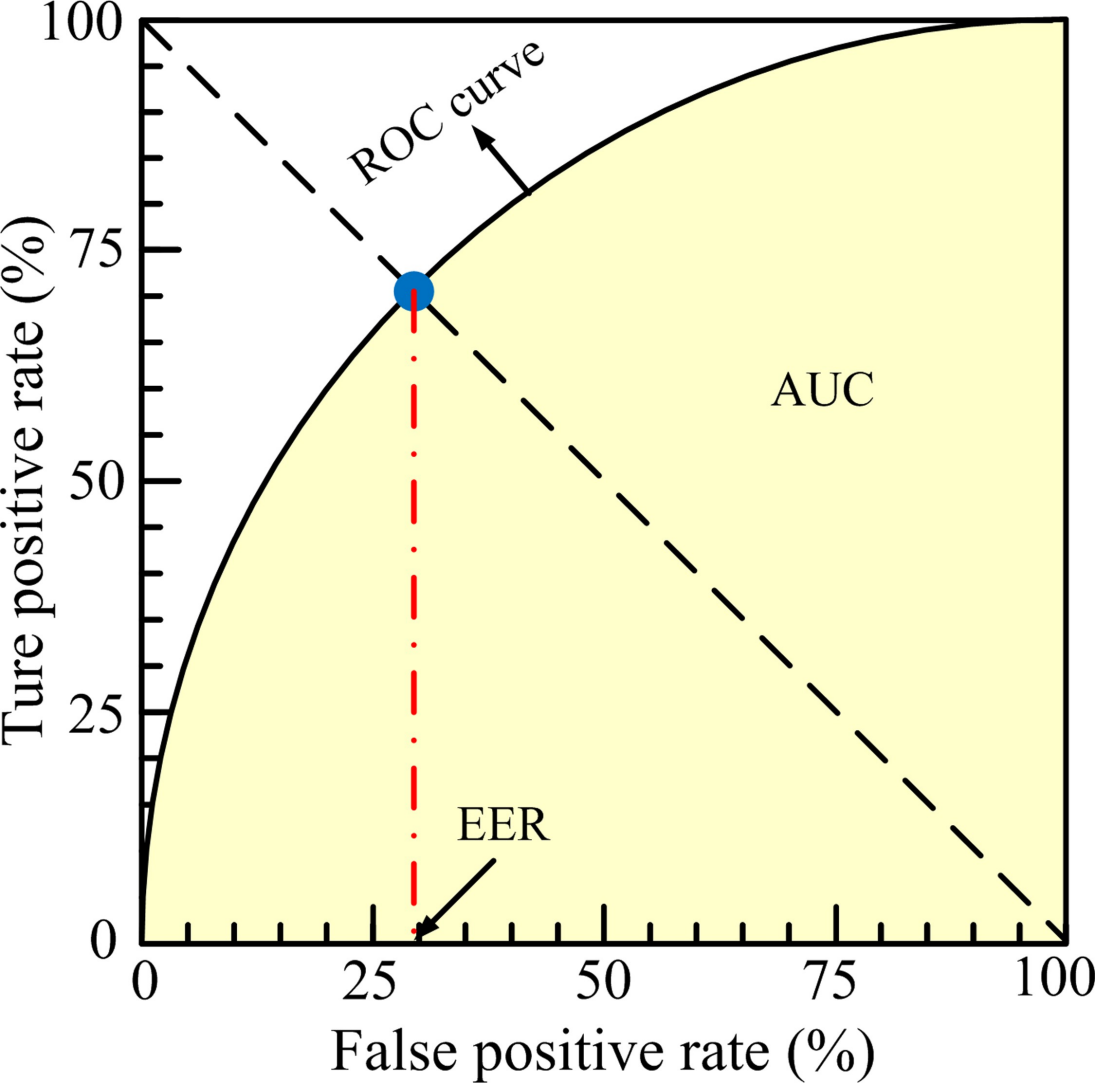
Relationship between AUC and EER.

To measure the quality of our method, we do the related experiments and use the receiver operating characteristic (ROC) curve as an indicator. The ROC curve is plotted by giving different threshold values and computing the true positive rate (TPR) and the false positive rate (FPR). We compare our approach with the existing anomaly-detection algorithms through the area under the curve (AUC) and equal error rate (EER). Higher AUC and lower EER values indicate the better performance of anomaly detection. The graphic illustration between AUC and EER is presented in Fig 6.

### Datasets description

CUHK Avenue Dataset contains 16 training videos and 21 testing videos with 360 × 640 pixels resolution obtained from Campus Avenue at the Chinese University of Hong Kong. The pedestrians coming in and going out of the building are regarded as normal events, and the abnormal events are throwing objects, running, loitering, and so on.

UCSD Dataset includes two subsets, Ped1 and Ped2, collected by the University of California San Diego. Ped1 consists of 34 training scenes and 36 testing scenes with 238 × 158 pixels resolution, and Ped2 comprises 16 training scenes and 12 testing scenes with 360 × 240 pixels resolution. In all normal cases, the people walk on the sidewalk. The abnormal videos contain bicycles, skateboarders, wheelchairs, and vehicles crossing pedestrian areas.

### Training details

For the training details of our algorithm, we choose Adam [31] to optimize the model parameters. The model adopts a random clip of five sequential frames normalized to [-1, 1] in the training phase. In addition, we set T to 4, and the mini-batch size is also 4. Concerning the generator and discriminator, the learning rates are assigned to 0.0001 and 0.00001 for grey-scale datasets, corresponding to 0.0002 and 0.00002 for color-scale datasets. For different datasets, the coefficient factors *α*_*int*_, *α*_*gd*_, *α*_*rgb*_, and *α*_*adv*_ were slightly different.

### Experimental results

#### Results on the Avenue dataset

**Fig 7:**
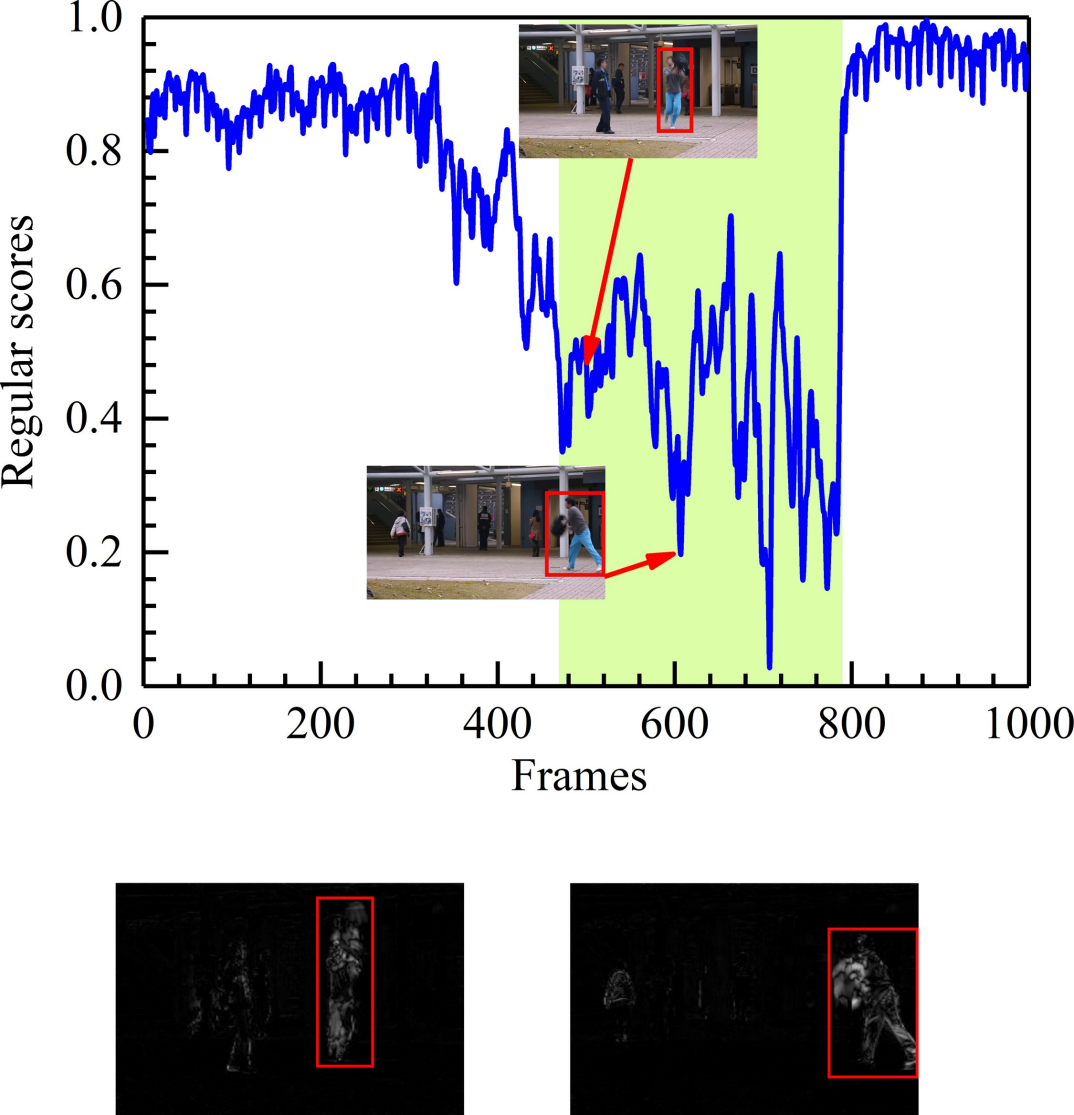
Frame-level evaluation results on the 5th test video of Avenue. (a) Relationship between the test video frames and the regular score. (b) Difference between the ground truth and the corresponding generated frame.

For a detailed description, in Fig 7, some events are shown as the anomaly detection results from the fifth test video in the CUHK Avenue dataset. Fig 7A displays the relationship between the test video frames and the regular score. The green blocks denote the ground truth abnormal region, and the blue line represents the regular score of every frame. Higher regular scores indicate normal events. On the contrary, the lower regular scores matching the green area are anomalous events (e.g., throwing the bag). Fig 7B presents the difference (labeled with a red rectangle) between the ground truth and the corresponding generated frames. When running the proposed algorithm, the model has learned prior knowledge and predicts what will happen next. Under the campus avenue scene, the training samples are all normal clips of walking persons. Once the test events do not match the appearance and motion characteristics of the training samples, it will generate a big difference between the generated frame and the ground truth.

#### Results on the UCSD dataset

**Fig 8:**
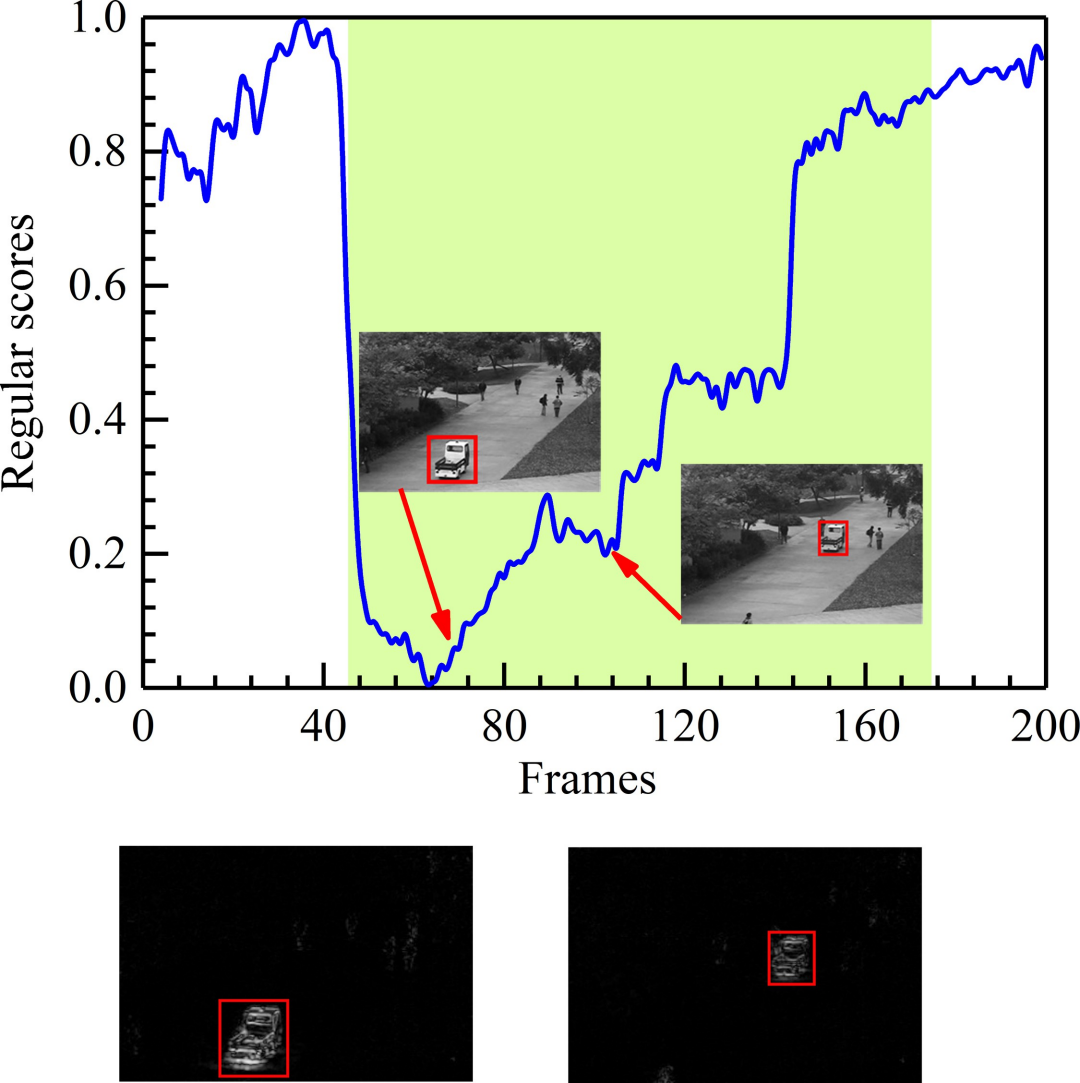
Frame-level evaluation results on the 20th test video of UCSD Ped1. (a) Relationship between the test video frames and the regular score. (b) Difference between the ground truth and the corresponding generated frame.

**Fig 9:**
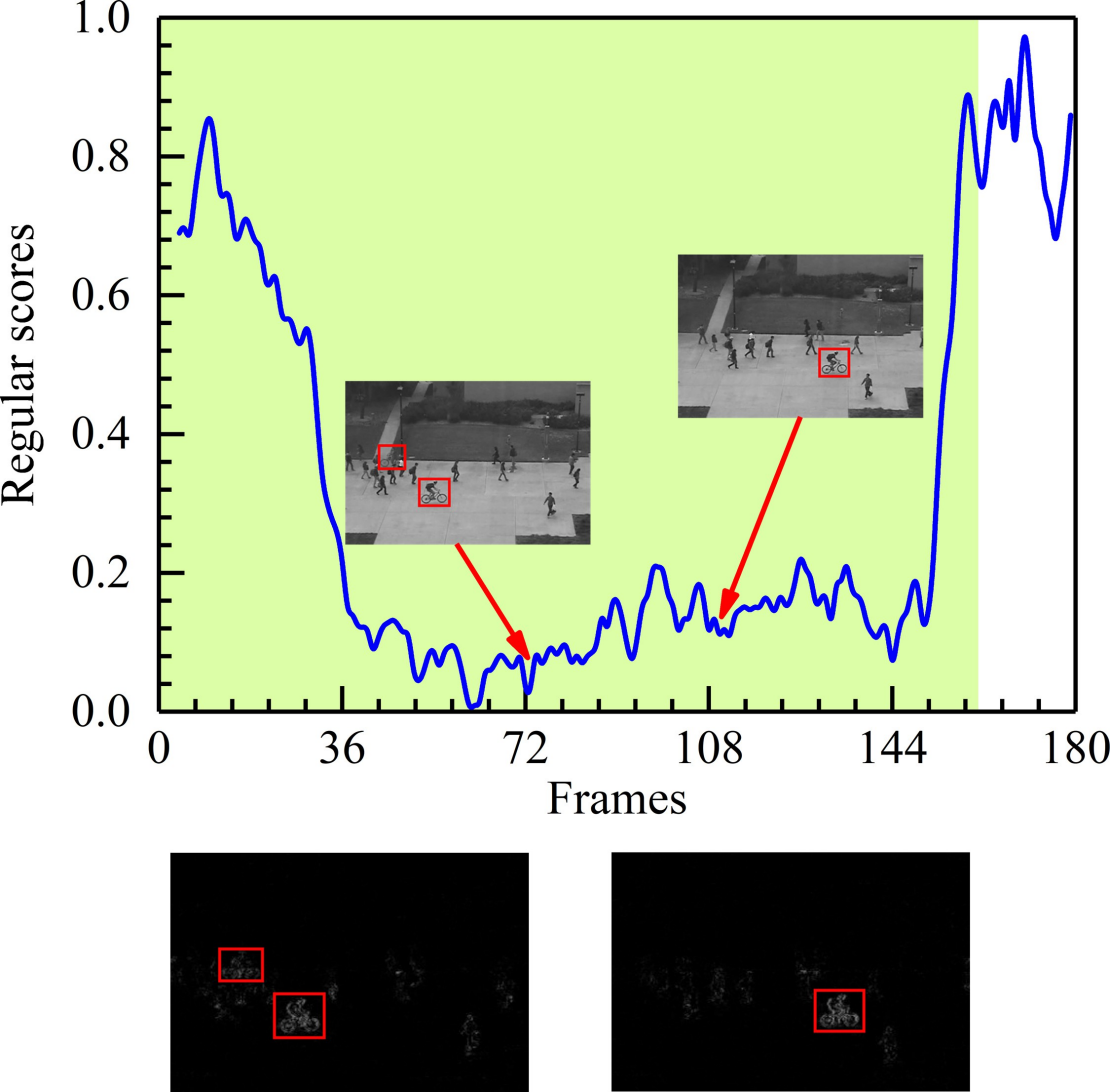
Frame-level evaluation results on the 6th test video of UCSD Ped2. (a) Relationship between the test video frames and the regular score. (b) Difference between the ground truth and the corresponding generated frame.

The size and shape of objects may vary on account of the position and direction of the camera. Specifically, Figs 8 and 9 display the experimental results of detecting anomalies from different camera directions on the UCSD Ped1 and Ped2 datasets. The meanings of these figures are similar to those in Fig 7. Shown here, in Figs 8A and 9A, the lower regular scores represent the abnormities (e.g., the car in the UCSD Ped1 20th test video and the cyclists in the UCSD Ped2 6th test video). Higher regular scores are consistent with normal behaviors. Just as Figs 8B and 9B depict, objects near the camera look more prominent than those far from the camera, even though they are the same objects. We find that abnormal events can be easily detected in different situations. Through analyzing the experimental results, it is evident that our method performs well with the different scales of spatial features because it uses the strengths of HDC to focus on the corresponding feature information.

## Discussion

By analyzing the corresponding experimental results of different datasets, Table 1 shows a quantitative comparison between our method and other deep learning approaches for frame-level AUC. We find that the AUC values of our method are higher than that of the other approaches, demonstrating great detection ability. Due to the evident capability for anomaly detection based on a prediction network, we set the literature [19] as the baseline during the testing phase. In detail, our approach raises 2.0%, 1.2%, and 1.6% for UCSD Ped1, UCSD Ped2, and CUHK Avenue datasets compared with it. We can see that Ped1 datasets improve higher AUC values than baseline [19]. The reason lies in that the reconstruction module of our method is strong enough to overcome defects in the underlying noise of the Ped1 data. Moreover, our method gets better results than these approaches [9,10] because of fusing an improved prediction module in our model. This prediction module integrates HDC and DB-ConvLSTM strategies to widen the gap between normal and abnormal events and improve the quality of predicted frames from the space and time dimension.

**Table 1:** Comparison of frame-level AUC values on different datasets.

Methods	AUC(%)		
	UCSD Ped1	UCSD Ped2	Avenue
Conv-AE [6]	81.0	90.0	70.2
STAE [9]	87.1	88.6	80.9
ConvLSTM [15]	89.9	87.4	80.3
ConvLSTM-AE [16]	75.5	88.1	77.0
MGFC-AAE [17]	85	91.6	84.2
Unmasking [32]	68.4	82.2	80.6
AnomalyNet [33]	83.5	94.9	86.1
Baseline [19]	83.1	95.4	84.9
IPR [10]	84.7	96.3	85.1
Proposed method	85.1	96.6	86.5

In addition, we also choose EER as the evaluation metric to demonstrate the superiority of our approach. Table 2 shows the experimental results obtained from our method and other algorithms. Compared with different techniques, we find that our method reaches a lower EER except for ConvLSTM [15] (UCSD Ped1) and AnomalyNet [33] (UCSD Ped2).

**Table 2:** Comparison of EER values on different datasets.

Methods	EER (%)		
	UCSD Ped1	UCSD Ped2	Avenue
Conv-AE [6]	27.9	21.7	25.1
STAE [9]	18.3	20.9	24.4
ConvLSTM [15]	12.5	12	20.7
MGFC-AAE [17]	20	16	22.3
AnomalyNet [33]	25.2	10.3	22
Baseline [19]	24	12	21
Proposed method	22.3	10.5	19.4

### Ablation studies

#### Comparing different parts

To evaluate the performance of each part of the prediction module for our method, we performed an ablation study for a different part. Specifically, three variants (i.e., prediction module only with HDC, ConvLSTM, and DB-ConvLSTM) were trained to access the effects of anomaly detection. Table 3 presents the AUC values computed from the variants with different parts on the datasets. It can be seen that the variant with all parts reaches the best results than those with fewer parts, which indicates the importance of taking full advantage of the spatio-temporal features for anomaly detection. In detail, the HDC can capture more comprehensive multi-scale spatial characteristics, and the DB-ConvLSTM can obtain temporal information.

**Table 3:** Effect of different parts for prediction module on different datasets.

Components	AUC(%)		
	UCSD Ped1	UCSD Ped2	Avenue
HDC	83.9	95.8	85.5
ConvLSTM	83.7	95.5	85.2
DB-ConvLSTM	84.1	95.7	85.6
HDC& DB-ConvLSTM	85.1	96.6	86.5

#### Robustness to noise

**Fig 10:**
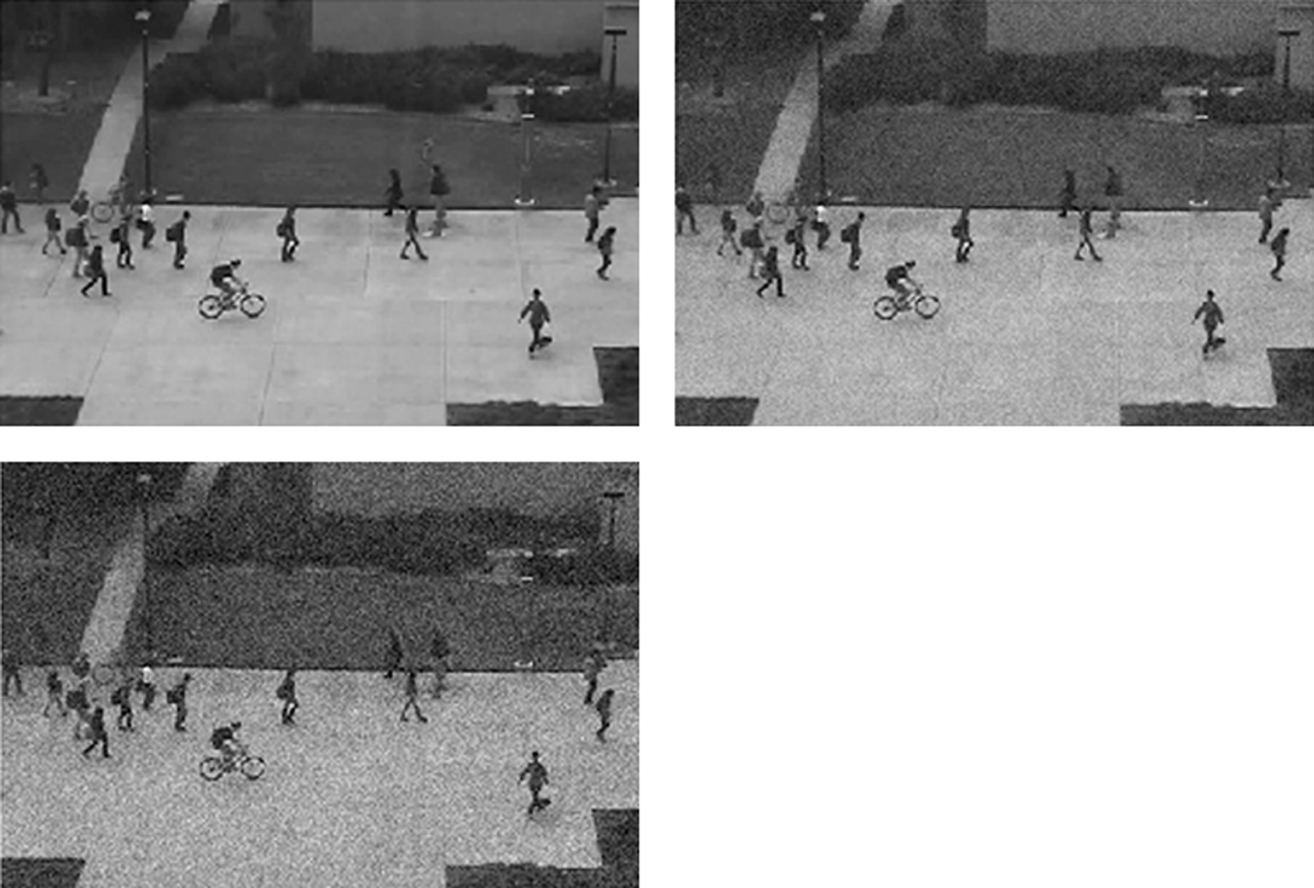
Frames with different Gaussian noise.

**Fig 11:**
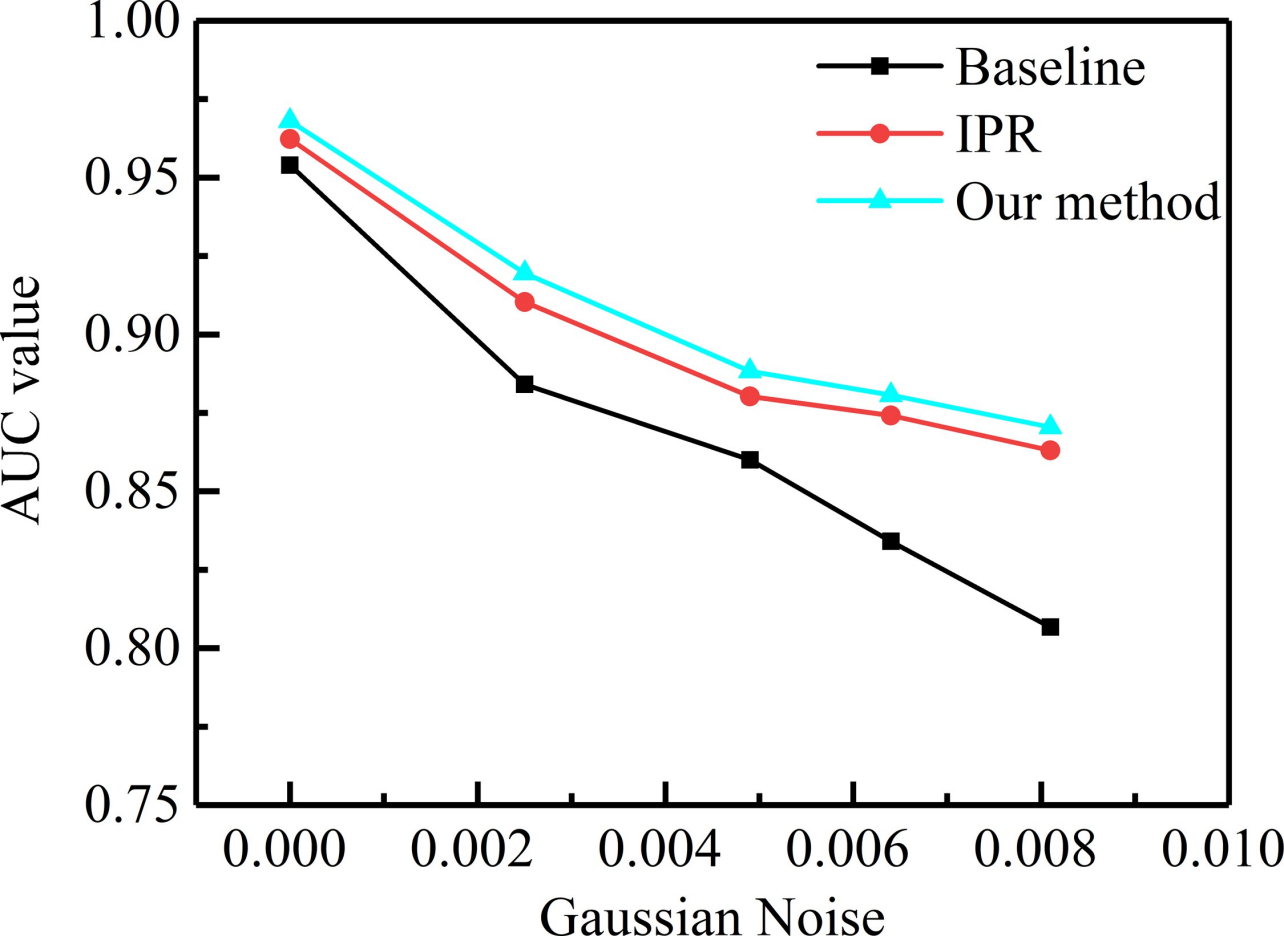
AUC value of different methods in different Gaussian noise.

To prove the anti-noise performance of our method, we added the Gaussian noise with different variances to the datasets. For intuitively presenting the visual images, video frames from the UCSD Ped2 dataset with variances of 0.03 and 0.06 are shown in Fig 10. We used these data to experiment with different methods. The results are illustrated in Fig 11 for the varying curve of AUC with different Gaussian noise values. Obviously, the AUC values of all methods steadily decrease as Gaussian noise increases. We find that our approach has better robustness to noise than the baseline [19]. The main reason is that the reconstruction module with the strong generalization ability is connected after the improved prediction module. Thus, our method can overcome the problems caused by the noise and effectively improve the quality of the generated frame.

## Conclusion and future work

In this paper, since the quality of future frame prediction is vital for anomaly detection, we propose a practical prediction module by adding HDC and DB-ConvLSTM strategies to capture more detailed multi-scale spatial features and temporal information of normal events. Furthermore, we integrated the reconstruction module after the prediction module to improve the entire model’s noise immunity due to the lousy anti-noise performance. We carried out the experiments on some publicly available datasets to verify the proposed model. The experimental results show that the AUC values were 85.1%, 96.6%, and 86.5%, corresponding to UCSD Ped1, Ped2 datasets, and CUHK Avenue. Compared with state-of-the-art approaches, our method does well in detection accuracy through qualitative analysis and quantitative comparisons.

The proposed method does not limit the type of abnormality, and it can achieve the general detection of different abnormal behaviors in a specific scenario. Therefore, our approach can be well applied to many video surveillance scenes. However, the proposed model depends on the completeness of the training data of the scenarios, implying that the data should contain all normal events. In the future study, we plan to extend existing datasets to include as many surveillance video scenes as possible to address smart-city and public-security issues.
